# Proteus Syndrome: a difficult diagnosis and management plan

**Published:** 2014

**Authors:** MD Popescu, G Burnei, L Draghici, I Draghici

**Affiliations:** * ”Carol Davila” University of Medicine and Pharmacy, Bucharest; ** Department of Pediatric Surgery, “Maria Sklodowska Curie” Clinical Emergency Hospital for Children, Bucharest; *** Department of General Surgery, “Sf. Ioan” Clinical Emergency Hospital, Bucharest

**Keywords:** Proteus syndrome, overgrowth, connective tissues abnormalities, cerebriform lesion

## Abstract

**Rationale.** Proteus Syndrome (PS) is an extremely rare congenital pathology that causes overgrowth of multiple tissues, in particular bone and fat, following a mosaic pattern. The estimated incidence is of less than 1 per 1,000.000 live births and represents a significant challenge to the pediatric and orthopedic surgeons in order to establish a diagnosis and to elaborate a management plan.

**Objectives.** We had the opportunity of treating many children who were afflicted by overgrowth syndromes and have been previously misdiagnosed as Proteus Syndrome in our department of pediatric and orthopedic surgery of “Maria Sklodowska Curie” Clinical Emergency Hospital for Children. This study helped us develop a diagnostic for these patients and report the first case of a confirmed PS in Romania.

**Methods and Results.** We report the case of a 5-year-old white male who is in the attention of the clinic since birth. He presented with multiple overgrowth bone segments, fatty subcutaneous or intraabdominal tumors and other connective tissues abnormalities. All the tests performed confirmed the diagnosis of PS at the age of 4 and the management is still to be decided.

**Discussions.** We followed the latest diagnostic indications and the patient fulfilled the general and specific criteria. The treatment is still in progress and it represents a challenge for the multidisciplinary medical team.

**Abbreviation** Proteus Syndrome = PS

## Introduction

Proteus Syndrome is an extremely rare medical entity that has first been delineated by Cohen and Hyden in 1979 and, four years later, received the name after the Greek god Proteus, who was capable of assuming multiple forms. The diagnostic criteria were clearly established in 2004 by Turner and the responsible gene, AKT1, was discovered in 2011, confirming the genetic nature of the syndrome. The estimated incidence is of less than 1 per 1,000,000 live births, with a male/female ratio of 1, 9/1 [**1**,**2**]. A sporadic disorder causes an overgrowth of skin, bones, fat tissues, muscles and lymphatic vessels. All the lesions appear to be distributed in a mosaic manner and have a progressive evolution. It is estimated that 120 persons with PS are currently alive worldwide, and, in this article, we present the first confirmed PS case in Romania [**3**].

**The presentation of the case**

**Fig. 1:**
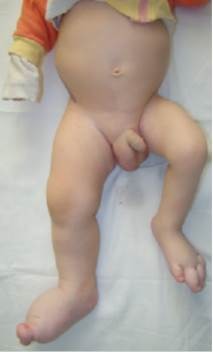
Disproportionate, asymmetric overgrowth of the lower limbs specific for PS

We report the case of a 5-year-old white male, born by normal vaginal delivery at 40 weeks’ gestation to normal healthy parents after an uneventful pregnancy. Birth weight, height and head circumference were 3500g, 53 cm and 32 cm, respectively. From birth, the patient presented with a disproportionate, asymmetric overgrowth of the lower limbs, macrodactyly of the feet bilaterally, right limb bigger than left, having a length difference of 3 cm. Other important findings include important bilateral lower extremity overgrowth of the feet, right calf and thigh, and a 6th supernumerary right toe (**Fig. 1**).

**Fig. 2:**
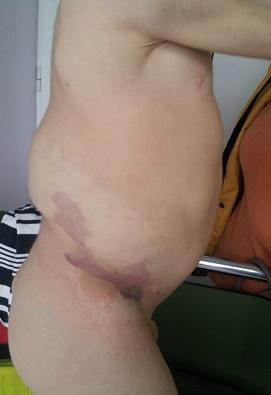
Pigmented epidermal nevi

An abdominal wall mass was palpable from the hepatic dome to the iliac crest, having a soft consistency, and we noticed pigmented epidermal nevi located on the right hemiabdomen and thigh (**Fig. 2**).

The facial phenotype was normal and there was no similar case in the family. The X-rays and MRI exams showed overgrown long bones of the right femur, tibia and fibula, and bilaterally of the metatarsal bones and phalanges. The abdominal wall mass had the aspect of a lymphangioma on the X-rays and the overgrowth soft tissue of the right thigh, calf and feet was represented by fatty overgrowth and lymphatic malformations disposed within muscles and subcutaneously. We also noticed a mild hepatosplenomegaly. The patient satisfied the general and specific criteria for PS as listed in **Table 1**.

**Table 1:** Diagnostic criteria for Proteus Syndrome

**General criteria**	
1.Mosaic distribution of lesions (there are unaffected corporeal segments)	
2.Progressive course of the symptoms	
3.Sporadic occurence of the pathology (no similar cases in the family)	
**Specific criteria**	
*Category A*	
Presence of the cerebriform connective tissue nevus	General criteria
	+ 1 A criteria
	=
	Proteus Syndrome
*Category B*	
1.Epidermal nevus	General criteria
2.Asymmetric, disproportionate overgrowth (limbs, viscera, hyperostotsis of the skull, megaspondylodysplasia, hyperostosis of the external auditory meatus)	+ 2 B criteria
3.Specific tumors before the 2nd decade (parotid monomorphic adenoma, ovarian cystadenoma)	=
	Proteus Syndrome
*Category C*	
1.Dysregulated adipose tissue (lipomas, regional lipohypoplasia)	General criteria
2.Vascular malformations (capillary, venous, lymphatic)	+ 3 C criteria
3.Lung cysts	=
4. Facial phenotype (Dolichocephaly, long face, downslanting palpebral fissures, minor ptosis, low nasal bridge, wide nares, open mouth at rest)	Proteus Syndrome

At 1 year of age, the patient presented a progressing lower limb discrepancy of 6 cm, an enlargement of the disproportionate, asymmetric overgrowth of the abdominal wall mass and the presence of a new soft tumor on the left buttock. In the anterior part of the calf, two hard masses were identified, described by the ultrasound as fat necrosis. The latter imagistic examinations showed many intraabdominal cysts in the peritoneal cavity, pelvic region, probably lymphangiomas and increase of the hepatosplenomegaly. Lab works confirmed an anemic syndrome and a treatment has been prescribed. The mental development was normal corresponding to age.

At 3 years, the patient has been subjected to a colonoscopy due to daily rectal bleedings and a persisting anemic syndrome. The results showed a lymphonodular hyperplasia of the colon.

**Fig. 3:**
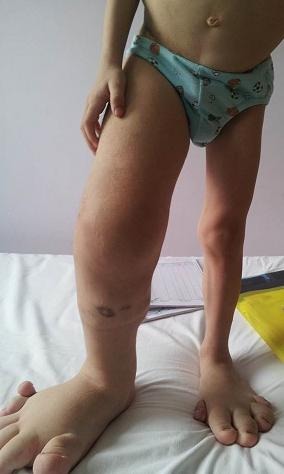
Age 4, length difference of 9 cm between the right and left limb

At the age of 4, the limb length discrepancy was 9 cm (**Fig. 3**).

**Fig. 4:**
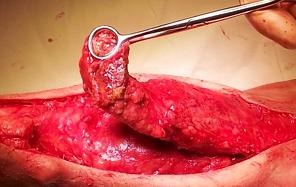
Excision of an important fatty overgrowth and lymphangioma from the posterior part of the right calf

The patient had a normal mental development and was socially integrated, attending kindergarten. The orthopedic surgeons decided to perform a procedure on the right calf in order to diminish the difference of thickness and to prevent further length discrepancy of the two lower limbs. Under general anesthesia, an excision of an important fatty overgrowth and lymphangioma of the posterior part of the right calf was performed (**Fig. 4**), ablation of the 6th right toe and permanent epiphysiodesis of the distal tibia and fibula.

Some of the biopsy tissues were sent to the National Institute of Health USA for the molecular confirmation of the PS. The pathologic examination confirmed the presence of acanthosis, hyperkeratosis and a highly collagenized connective tissue, intramuscular lymphangiomas and fat hyperplasia with small necrosis areas.

Nowadays, the patient is socially integrated and the lower limb length discrepancy is of 7 cm. The quality of life does not impose a surgical approach of the abdominal wall mass or of the intraperitoneal cysts. The rectal daily bleedings and the anemic syndrome persist and require further investigations.

## Discussions

PS is an extremely rare disorder characterized by multiple tissues overgrowth, especially bone and fat, vascular malformations, cerebriform lesions or epidermal nevi. Due to its rarity and high variability, it has been difficult to establish sensitive and specific diagnostic criteria. Misdiagnosis of PS has been common before the publication of the diagnostic criteria, first in 1999, by Biesecker, completed by Turner in 2004 (**Table 1**) [**2**]. Patients must satisfy both the general criteria and the specific categorical criteria in order to be conferred the diagnosis of PS. Those who do not meet the stringent diagnostic criteria for PS have been reclassified as patients with overgrowth non Proteus Syndrome [**3**,**4**]. In our case, the patient fulfilled all the general criteria (mosaic distribution, progressive evolution and sporadic occurrence), together with 3 specific criteria form the B category, presence of the epidermal nevi, asymmetric disproportionate overgrowth of two limbs and splenomegaly.

Patients affected by PS have a unique clinical presentation due to the mosaic pattern of the disease distribution. Two genetically different populations of cells that originated from a zygote with uniform cells can be found in their organism. The mutated cells distribute randomly into the body and create different clinical phenotypes of PS [**2**,**4**]. Our patient had multiple lesions affecting the lower limbs, abdomen wall, lymphatic system, and intraperitoneal findings. In 2011, the responsible gene mutation has been identified at the level of AKT1 gene, confirming the molecular basis of the syndrome [**1**,**2**].

Overgrowth of the tissues in PS is not only asymmetric and disproportionate but also progressive, distorting and relentless. The lower limb length discrepancy of our patient progressed in 4 years from 3 to 9 cm, imposing a surgical approach in order to stop this distorting process.

The last general criteria, a sporadic occurrence, refer to the fact that the patient was the only sibling affected by this syndrome. There are references in literature of at least three PS patients who have given birth to unaffected offspring [**2**].

Guidelines for the evaluation and management of the patients with PS have been elaborated including clinical photos, skeletal X-rays of the affected body areas, CT scans, MRI and other consults such as dermatology, neurology, ophthalmology and hematology [**2**,**5**]. About 20% of the PS patients have had premature deaths resulting from pulmonary embolism, postoperative complications and pneumonia. The risk of a deep venous thrombosis has to be considered when managing such a patient. The advantages and risks of a surgical procedure must be carefully evaluated and all precautions must be taken if an intervention is absolutely necessary [**2**,**4**]. In our case, antithrombotic prophylaxis was performed before and after surgery and the treatment and evolution was supervised by a cardiologist.

The last issue to discuss is the important psychosocial impact that this syndrome has upon the affected children and their parents. The pathology is a rare, progressively disfiguring condition and becomes a social stigma in many cases [**2**,**4**]. Our patient is integrated in the social life, attends kindergarten. Unfortunately, the child and his parents are not under psychological counseling and, in our country, there are no support groups. His mental development was normal and there were no life threatening lesions present at the time of his last evaluation.

## Conclusions

Following the natural relentless evolution of the pathology, we recommend frequent clinical and imagistic follow-ups, in order to detect and avoid life threatening or distorting complications, such as progressive skeletal deformities, invasive lipomas and lymphangiomas, benign and malignant tumors. The dangers of deep venous thrombosis with pulmonary embolism limit the surgical approach to a minimum. A multidisciplinary team aware of these complications and their management is essential in order to provide the best optimal care for the patients with PS.

**Acknowledgement**

This paper is supported by the Sectoral Operational Program Human Resources Development (SOP HRD) 2007-2013, financed from the European Social Fund and by the Romanian Government under the contract number: POSDRU/159/1.5/S/137390

**Disclosures**

None
